# An Approach for Removing Redundant Data from RFID Data Streams

**DOI:** 10.3390/s111009863

**Published:** 2011-10-20

**Authors:** Hairulnizam Mahdin, Jemal Abawajy

**Affiliations:** 1 Faculty of Computer Science and Information Technology, University of Tun Hussein Onn Malaysia, Parit Raja, Batu Pahat, Johor 86400, Malaysia; E-Mail: hairuln@uthm.edu.my; 2 School of Information Technology, Deakin University, Waurn Ponds, Victoria 3216, Australia

**Keywords:** RFID, duplicate reading, automatic identifications, data filtering

## Abstract

Radio frequency identification (RFID) systems are emerging as the primary object identification mechanism, especially in supply chain management. However, RFID naturally generates a large amount of duplicate readings. Removing these duplicates from the RFID data stream is paramount as it does not contribute new information to the system and wastes system resources. Existing approaches to deal with this problem cannot fulfill the real time demands to process the massive RFID data stream. We propose a data filtering approach that efficiently detects and removes duplicate readings from RFID data streams. Experimental results show that the proposed approach offers a significant improvement as compared to the existing approaches.

## 1. Introduction

In many applications such as manufacturing, distribution logistics, access control, and healthcare, the ability to uniquely identify, real-time product track, locate and monitor individual objects is indispensable for efficient business processes and inventory visibility. The use of radio-frequency identification (RFID) technology has simplified the process of identifying, tracking, locating and monitoring objects in many applications. RFID uses radio-frequency waves to transfer identifying information between tagged objects and readers without line of sight, providing a means of automatic identification, tracking, locating and monitoring. Many organizations are planning to or have already exploited RFID to achieve more automation, efficient business processes, and inventory visibility. For instance, after implementing RFID system, Wal-Mart reportedly reduced out-of-stocks by 30 percent on average at the selected stores.

While RFID provides promising benefits in many applications, there are issues that must be overcome before these benefits can be fully realized. One major hurdle to be fully addressed is the problem of duplicate readings generated dynamically in very large data streams. The problem of duplicate readings in RFID is a serious issue that needs an efficient approach to solve it [1]. While the current RFID reader accuracy is improving, redundant data transmission within the network still occurs in RFID systems. Some of the factors that contribute to the duplicate data generation include unreliability of the readers and duplicate readings generated by adjacent readers. A reading is defined as duplicate when it is repeated and does not deliver new information to the system. Duplicate readings unnecessarily consume system resources and impose traffic burdens on the system. Because RFID-enabled applications primarily use RFID data to automate business processes, inaccurate and duplicate readings could misguide application users [2]. Therefore, RFID data must be processed to filter out duplicates before an application can use it.

Although, there are many approaches in the literature to filter duplicate readings [3–6], most of the existing approaches focus on data level filtering. They also tend to have high computation costs and they do not reduce much the transmission overhead. Moreover, they tend to focus on a single RFID reader system whereas we focus on a system with multiple readers. Many applications used multiple readers for different purposes including increasing the reading ability [7]; reading objects passing by different doors at the warehouse [8]; and supply chain management [9]. However, the use of multiple readers creates duplicate readings, where readers perform readings on the same tagged object.

In this paper, we propose an approach to detect and remove duplicate readings at a reader level. The contribution of this paper can be summarized as follows: (a) an approach to detect and remove duplicate readings from a RFID data stream, and (b) experimental analysis of the proposed approach and comparison with sliding windows-based approach [3] and few other approaches that were proposed in [5,10]. The results show that our proposed approach demonstrates superior performance as compared to the other baseline approaches. The proposed approach is an extension of our previous work [6] in two ways. First, unlike the previous algorithm that used two filters, in this algorithm the number of filters have been reduced from two to only one. Removing one of the filters reduces the time needed to filter duplicate readings without reducing the accuracy and efficiency of the filtering results. By reducing the number of tasks, the new algorithm increases the efficiency and makes the approach less complicated. Second, the current algorithm incorporates an enhanced landmark window in the duplicate filtering process. This landmark window will remove all data when a specific point is met.

The rest of the paper is organized as follows: Section 2 presents the background of the problem which includes system model and problem formulation. Section 3 discusses the related work. The proposed algorithm is described in detail in Section 4. Section 5 presents the performance analysis of the proposed algorithm. The results in this section show that the proposed approach significantly outperforms existing approaches. The conclusion and future directions is presented in Section 6.

## 2. Background

### 2.1. System Model

A typical RFID system consists of a transponder (*i.e.*, tag), which is attached to the object to be identified, an interrogator (*i.e.*, reader) that creates an RF field for detecting radio waves, the middleware and a backend database system for maintaining expanded information on the objects and other associated information. The middleware collects and processes the readings from readers for the use of enterprise applications and enterprise database. The process such as filtering and aggregation transform raw data into meaningful information for the application. The middleware also coordinates reader activities, ensures reliability in data transmission, improving network communications and allowing heterogeneous devices to collaborate together [11].

The tag is capable of storing the identifying information of the object to which it is attached and communicate the information via radio waves to an interrogator. RFID tags, based on their power sources, are generally classified as passive, active and semi-active tags. The active and semi active tags have their own power source whereas the passive tags forage power from the waves sent out by readers. The semi-active tags may also scavenge power from the readers. In this paper, we focus on passive tags. Relative to both active and semi-active tags, passive tags are very cheap and they are widely used in very large quantities in many applications such as supply chain management.

The RFID system is assumed to contain multiple networked RFID readers deployed to collaboratively collect data from tagged objects in their vicinity. The reading distance ranges from a few centimeters to more than 300 feet, depending on factors such as interference from other RF devices. The RFID readers query tags to obtain data and forward the resulting information through the middleware to the backend applications or database servers. The applications then respond to these events and orchestrate corresponding actions such as ordering additional products, sending theft alerts, raising alarms regarding harmful chemicals or replacing fragile components before failure.

### 2.2. Problem Overview

Duplicate readings exist at the reader level and at the data level [1]. Duplicate readings at the reader level exist when the tagged object is being read by more than one reader. This can happen when there are overlaps in the reading vicinity of multiple readers. In contrast, duplicate readings at the data level occur when an RFID reader keeps reading the same object repeatedly. This will occurs when the tagged object does not move from the reader’s vicinity. In this case, the useful readings are those that indicate the tagged objects entering and leaving the readers reading vicinity. The other readings between these two events are not useful as they do not indicate any new event in the system.

**Figure 1.**
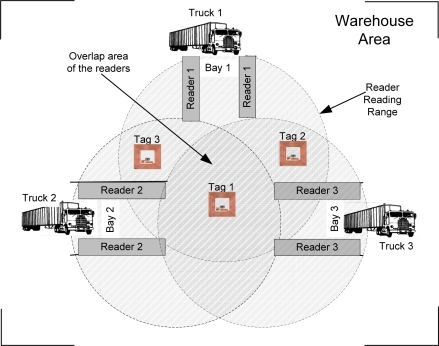
An RFID-enabled system for warehouse loading bay.

To illustrate the problem of duplicate readings at the reader level, we use an RFID-enabled system for a warehouse loading bay as shown in Figure 1. A warehouse can have a number of loading bays for pallets loading from a truck. Each bay is equipped with an RFID reader. When truck arrives, the pallet load into the warehouse will be automatically detected by the reader through their tags. Using this system, the pallet or an item does not need to be scanned manually, which saves time and labour costs. However, the reading range of reader 1 can overlap with the reading range of reader 2, as shown in the figure. This leads to duplicate readings [8]. If the readings on pallets in other reader vicinity are taken into account, the system will have an incorrect quantity derived from the readings. These duplicate readings need to be filtered out so that each reader for each bay will only report the pallets in the designated bay. Therefore, efficient methods for detecting redundant readers are of great importance for the development of wireless RFID networks.

In the environments where multiple RFID readers are deployed to monitor and track tagged objects, a duplicate reading due to overlapped readers is unavoidable and continues as long as the tag is in the overlapped regions. The duplicate readings results in unnecessary transmissions and consume network bandwidth. Enterprise applications are interested in a single reading copy, which makes appropriate data filtering mechanisms an important component of any RFID system.

## 3. Related Work

In this section, we will review the existing duplicate RFID data filtering approaches that are related to our work. Table 1 summarizes the existing duplicate RFID data filtering approaches. One way to remove data level duplicate readings is by using Extensible Sensor Stream Processing (ESP) [12]. In this approach, a query is imposed on the database records to retain an only single reading that represents other similar readings in a specified time frame. ESP uses predicates to get readings in a specified time frame, such as all readings today from 8.00 am to 8.00 pm and then corrects missed readings and detects outliers for a single sensor stream. Both these methods are proposed for cleaning sensor data streams. The complexity and nature of the RFID objects that dynamically move makes ESP unsuitable for RFID data streams [1].

RFID Cube [13] is a data warehouse based model to reduce duplicate readings in the RFID data stream. The approach is based on the assumption that the RFID objects tend to move and stay together and thus can be grouped together based on their locations. Both ESP [12] and RFID Cube [13] delay the filtering process (*i.e.*, they need to wait for the readings to be complete first like for the whole day before the filtering process can be started). This will cause wastage in network bandwidth and to store the duplicate readings. The Energy-Efficient In-Network RFID Data Filtering Scheme (EIFS) is proposed to filter duplicate readings in wireless sensor network [14]. The objective of the EIFS is to reduce the burden at the central processing and distributed the filtering task to the cluster head. In contrast, our aim is to identify a reader that can preserve the reading. Indeed, both approaches can be merged together to yield more interesting a result for future works.

Two possible ways of avoiding duplicate readings due to readers overlapping are to control the number of active readers at any given time [15,16] or to restrict the reading ranges of the readers [8]. An approach based on a radio frequency absorbing material to stop the RFID propagation from dispersing to neighboring reader’s area is discussed in [8]. The material is placed between readers to prevent a reader from reading neighboring tags. However, this solution is not feasible in most applications because of the design constraints and its high cost. An alternative approach is to systematically and selectively switch off readers to avoid redundant reading [15,16]. In the Redundant Reader Elimination (RRE) algorithm [15], the readers take turns reading the tags in their vicinity. The reader will write on each tag the total number of tag they have read along with the reader’s ID. The next reader can only overwrite the tag if the number of the tags it’s reading is greater. The reader that did not write on any tag will be switched off. The problem with the RRE algorithm is that it requires rewriteable tags to run. Rewriteable tags cost more than the passive tags and thus it is less commonly used in RFID deployments. It is also not practical to switch off readers in an environment where tags are not stationary since it requires a way to predict tag movements [1]. Another approach is to use the anti-collision algorithm such as MRFID [17]. MRFID is the enhancement of Time Division Multiple Access (TDMA) anti collision algorithm that takes into account the mobility of RFID reader. The reader will be put into a different slot to take turn in perform readings. However, this approach cannot be applied to applications that require all the readers to be operated at the same time such as shown in Figure 1.

An approach that uses sliding window to filter duplicate RFID readings is discussed in [3]. For stationary objects (e.g., items on the smart shelf), the objects will be read repeatedly going in the sliding windows repeatedly. The approach wastes resources as the effort for moving the item along the sliding windows is performed repeatedly. Another weakness of the sliding window approach is that the size can be not large enough to perform filtering correctly. One possible way to address this problem is using the landmark windows [18]. By using landmark windows, all readings that have been read will be kept in the window and do not need to go through the windows again. However, the problem is that the size of the landmark windows will be very big, which increases the processing time as well as use increased storage. Our approach does not suffer from this problem.

Approaches that explore the Bloom filter for filtering duplicate readings in RFID has recently emerged in the literature [4,5]. The main idea of the standard Bloom Filter (BF) [10] is to represent an element in a form of positive counter in a bit array of size *m* using *k* number of hash functions. All bits in BF are initially set to 0 and will be replaced by 1 when it is hashed by the element. To test whether an element is a member of a set, the element will be run through the same hash functions used to insert the elements into the array. The element is said to be the member of the set if all the bits in which the element was mapped to is positive. For each new element, the corresponding *k* bits in the array are set to 1. An approach that used the original Bloom filter to remove the duplicate is discussed in [4]. Two approaches were proposed in [4]: an eager and lazy approach that uses a Bloom filter to filter duplicate data. Generally, when a new reading comes at local reader, it will be inserted in the Bloom filter. The filter then will be sent to the central filter for update. The central filter coordinates readings from all the readers under its network. In the eager approach, the copy of the Bloom filter will be sent to every other reader to avoid the same reading from entering through them again. However it is too costly to update all the readers every time a new reading arrives. In the lazy approach, only a reader that sends a new reading will have new copy of the Bloom filter from the central filter. In our approach, we focus only on filtering at the central filter to preserve the reading only to the authorized reader. The work in [6] filters duplicate readings at a single reader. In contrast, the work presented in this paper takes into account multiple distributed RFID readers. Thus our work can be considered as complementary to the previous works.

## 4. RFID Data Stream Filtering Approach

**Figure 2.**
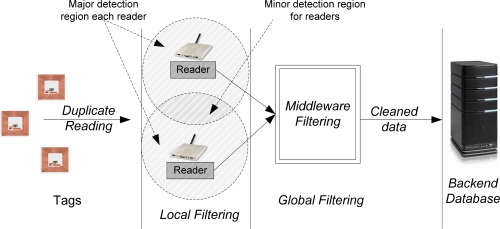
Multi-level RFID data filtering approach.

In this section, we discuss the proposed multi-level duplicate RFID reading filtering approach. The overall approach is shown schematically in Figure 2, where we classify reader detection ranges as a major or a minor detection region such that tags read inside the major detection area have 95% read rate while the tag read in the minor detection region have 5% read rate [19]. All incoming readings received by a given reader can be filtered locally at the reader (*i.e.*, local filtering) before the reading is sent to the middleware for another level filtering (*i.e.*, global filtering). The global filter at the middleware will filter duplicate readings among the readers before sending the data to the backend database or application.

Algorithm 1 shows the pseudo-code of the reader-level filtering, which we refer to as Comparison Bloom Filter (CBF). Note that the algorithm in Algorithm 1 is different from the one we discussed in [6]. Unlike the previous algorithm that used two filters, in this algorithm the number of filters has been reduced from two to only one. Removing one of the filters reduces the number of tasks needed to filter duplicate readings but does not reduce the accuracy and efficiency of the filtering result. By reducing the number of tasks it increases the efficiency and makes the approach less complicated. Also note that the algorithm in Algorithm 1 incorporates landmark window in the duplicate filtering process. Landmark window will remove all data when specific point is met.

**Algorithm 1.** RFID duplicate readings filtering algorithm.

**Algorithm CBF**
INPUT: C, TID
BEGIN
1: IF (Time == True) THEN
2: CBF[] = {0}
3: ENDIF
4: FOR (each incoming TID) DO
5: FOR (i = 1 TO k) DO
6: Pos ← Hashi(TID)
7: IF (CBF[Pos] == 0) | | (C > CBF[Pos]) THEN
8: CounterNum [i] ← Pos
9: ELSE
10: EXIT
11: ENDIF
12: ENDFOR
13: FOR (i = 1 TO k) DO
14: Pos ← CounterNum [i]
15: CBF[Pos] ← C
16: ENDFOR
17: ENDFOR
END CBF

The input to CBF is the reading count for each tag (C) and the tag identification (TID). The reading count (C) is needed to compare which reader have the higher reading on the tag (identify through TID). To do the comparison, CBF used an integer array, which allows its array counters to hold 8 bits data instead of 1 as in Bloom filter. The proposed algorithm also uses a landmark window which naturally suits the Bloom filter. We use a landmark window because RFID object movement cannot be easily predicted. Some of the objects may stay for a long time in the same area. Sliding windows are more suitable for applications that always have new readings that have not been recorded before.

In steps 1–3, the algorithm checks the time to remove all the readings. If the time is met, all CBF counters will be reset to zero. Next at step 4, a reading count for each tag is done at each reader and is sent with the TID to the filter which will run this algorithm. For steps 5–11, each incoming TID will be hashed and its condition checked. If the hashed counter value is 0 or smaller than C, the position will be retained in the filter. If one of the hashed counters did not satisfy the condition in step 7, the algorithm will exit (step 10) from all loop and start back to step 1 to receive a new reading. If all hashed counters satisfy the condition in step 7, steps 13–16 are carried out where the new value of C is stored in the CBF counter.

We give an example of inserting values in CBF from the hashing process. We have tag 1 from reader 1 with reading counts of 30. Tag 1 is hashed 3 times and the first hash return 0, the second hash return 1 and the third hash return 3. The value of 30 will be inserted to these hashed counters in CBF as shown in Table 2. To have the complete views, we give another example to show how CBF works. Initially, all the counters in CBF are set to 0. When new reading record arrives, CBF will only insert the count of the reading if the count is higher than current hashed counters values. Each time a reading is inserted into CBF, it means at that time the reader has the highest reading on that tag.

**Figure 3.**
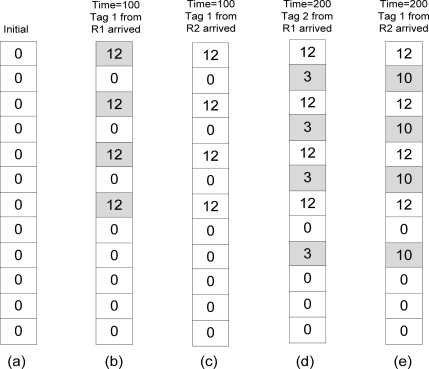
The state of CBF based readings in Table 3.

For example, refer to Table 3 and Figure 3. Table 3 lists the readings on tag 1 and tag 2 by two readers which are R1 and R2. Each reader will send the number of readings on their tag for every 100 s to the global filter. Initially all counters in CBF are set to 0 as shown in Figure 3(a). At time 100, R1 sends reading on tag 1 which is 12. The tag 1 is hashed *k* times and will be inserted in filters since there were no other readings previously.

All the hashed counters (shaded boxes) in CBF will be given value of 12 to represent the number of count as shown in Figure 3(b). Then reading on tag 1 by R2 arrived with number of readings 3. Tag 1 is hashed and returns the same counters like the previous reading. However this reading will be ignored by the filter because the number of readings by R2 on tag 1 is lower than the previous (Figure 3(c)). At time 200, R1 arrived with reading on tag 2 with number of readings 3. Tag 2 will be inserted in the filters since all the hashed counters returns 0 which means this is the new reading for tag 2 (Figure 3(d)). Now R1 has 2 objects (including tag 1). When reading from R2 arrives, it also reads tag 2 but with the higher reading than R1 did. The algorithm will insert the new number of readings on tag 2 by R2 in the filter and remove the previous reading of tag 2 by R1 (Figure 3(e)).

## 5. Performance Analysis

In this section, we use simulation to analyze the performance of the proposed duplicate reading detection and removal algorithm. We will first discuss the experimental setup and then present the results of the experiment and the discussion.

### 5.1. Experimental Setup

We model an RFID system with multiple readers and tags. The number of readers is set to two and we generated the data streams using Binomial distribution as in [19] and [20]. We increasingly vary the percentage of tags that resides in the overlapped reading range of the readers to test the robustness of the proposed algorithm. We compare the performance of the proposed algorithm against the sliding windows-based approach [3], the Bloom filter [10] and Decaying Bloom filter (DBF) [5] in terms of accuracy, time execution and false positive rate. We note that a slight modification is made to these algorithms to fit in with the problem that we are solving. We compare it with Decaying Bloom filter (DBF) [5].

### 5.2. Results and Discussions

#### 5.2.1. False Positive Rate as a Function of Hashing Function

In this experiment, we want to analyze the false positive rate (FPR) of CBF. A false positive occurs in CBF when a reading is detected incorrectly as a duplicate in the same window. We perform this experiment to find out the ratio of array size m to the number of readings n along with number of hash functions *k* that will return the lowest FPR. The result from this experiment will be used to set the parameters in the next experiments.

**Figure 4.**
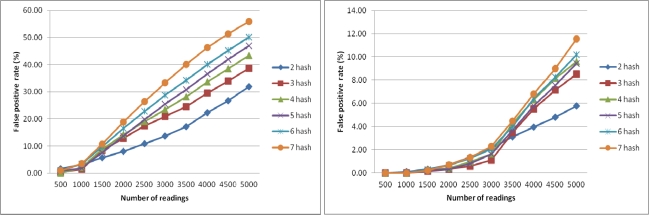
FPR of CBF as a functions *k* with counter size m = 5,000 (**left**) and m = 15,000 (**right**).

Figure 4 shows the FPR of CBF using different number of k with counter size m = 5,000 (left) and FPR of CBF using different number of hash functions *k* with counter size m = 15,000 (right). The number of readings varied from 500 to 5,000 with increment of 500 for each sample. Each sample is tested with different number of hash function k from 2 to 7. FPR is at the lowest when the number of readings is 500 and k is 7. The results show that the lowest FPR is achieved when the number of reading is less than 1,000 and the number of hash function is 7. In Figure 4 (right), when the size of sample is less than 1,500, FPR approaches almost zero percentage. Based on this result we conclude that to get the lowest FPR possible for CBF, the counter size m must be 10 times bigger than the number of readings with the number of hash functions is 7. We used this finding when running CBF for the next experiments to get the best results.

#### 5.2.2. Comparative Analysis of False Positive rate

**Figure 5.**
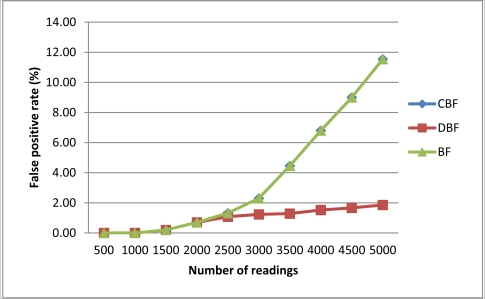
Comparison of FPR between Bloom filter approach.

In this section, we compare the proposed approach (*i.e.*, CBF) with the standard Bloom filter-based algorithm (BF) and the DBF approach. Figure 5 presents the FPR for each filtering algorithm. On average CBF and Bloom filter have the higher FPR compared to DBF because they use landmark windows that can become ‘full’ when the number of readings increases.

A ‘full’ Bloom filter is a situation where many of the counters in the filter has been hashed and hashing new elements returns on all hashed counters. In contrast DBF on average have lower FPR because they used sliding windows that always removed old readings from its counter. By this DBF cannot be easily becoming full. However, this does not mean DBF is better than CBF because as the result shown, we just need to choose the right filter size for CBF to get low FPR. The result from this experiment supports the conclusion we derived from previous experiment where CBF needed a filter size that is 10 times bigger than the number of readings.

#### 5.2.3. Rate of Unfiltered Duplicate Readings

In this experiment, we investigated the rate of unfiltered duplicate by CBF, Baseline, DBF and Bloom filter. This experiment measure the percentage of readings that are not being filtered correctly. Filtering correctly means that only readings that have the highest count on the tag will be inserted into the filter. For this experiment we generate 200 tags readings for two readers. The number of overlapped readings will be varied from 5% to 50% for each data set. Tags that are located in the major detection region will have 80% read rate while the minor detection region will have a 20% read rate. The reading cycle will be repeated for 10 times. The overlapped readings were scattered randomly in the data stream.

**Figure 6.**
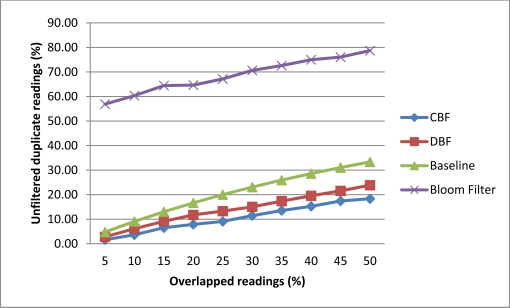
Percentage of unfiltered duplicate readings.

Figure 6 shows that CBF performs better than Baseline, DBF and Bloom filter in filtering the duplicate readings. CBF has the lowest unfiltered rates compared the other algorithms. The highest is the Bloom filter [10]. This is because Bloom filter could not store the data on the number of readings and reader ID which is needed to perform this task correctly. The sliding windows approach has the problem of filtering correctly when duplicate readings were scattered or skewed in the data stream. When the readings are scattered there are readings that cannot be compared with each other in the same windows. DBF also has the same problem as the Baseline approach because it is based on the sliding windows. CBF also performs better than other algorithms where on average it accuracy did not affected much with the additional of overlapped readings compared to others.

### 5.2.4. Execution Time Analysis

**Figure 7.**
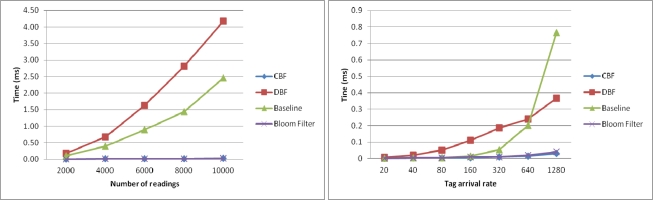
Time execution comparison to filter duplicate readings.

In this section, we examine the execution time of the algorithms to filter the duplicate readings as a function of number of readings and as a function of the tag arrival rate. Figure 7 shows the execution time of the algorithms to filter the duplicate readings as a function of number of readings (left) and as a function of the tag arrival rate (right). Figure 7 (left) shows the results the execution time to filter the duplicate readings in the first windows for every algorithm. In the experiment, the size of the sliding windows is set to be at the same size as the number of readings.

Thus, each approach will only use one sliding windows to process the readings. In this experiment, DBF took longer time than Baseline to complete the filtering. This is because the number of counters it has to maintain is very big to match with the number of readings. Every counter need to be decreased by 1 each time new reading is coming to simulate the sliding windows. CBF and Bloom filter took the least time to perform the filtering.

Figure 7 (right) shows the execution time of the algorithms to filter the duplicate readings as a function of the tag arrival rate (right). In the experiment, the data set generated have different number of reading arrivals per cycle is set to 20, 40, 80, 160, 320, 640 and 1,280. As can be seen, CBF performs better in terms of execution time than other algorithms. Baseline, which is based on the sliding windows approach, takes more time to execute, especially when the readings have high arrival rates per cycle. This is because it has to go through along the windows that become bigger with the increase of tag arrival rate for each new incoming readings. The same thing occurs in DBF. This is different from CBF where the arrival rate does not have an exponential effect on its time processing. Unlike Baseline and DBF, CBF does not have to go through along the windows to check for the duplication. The operation of hashing the tag ID and checking its existence in the filter is a constant operation O(n). For the Bloom filter the performance is equal with CBF. However as the previous result shows, it has very high unfiltered duplicates which make it unsuitable to perform this task.

## 6. Conclusions and Future Directions

In this paper, we have studied the RFID data duplication problem and proposed a new approach which is based on a Bloom filter. We compared the performance of the proposed approach with several existing approaches. The results show that proposed approach has a low false positive rate which illustrates the improved correctness of the filtering process. Our approach is more efficient in terms of time and memory usage whereas the results from the experiment show it to have better execution time than the others. In this paper we also demonstrated how sliding windows are not suitable in RFID scenarios where the tag’s movement cannot easily be predicted. The sliding windows consumes too much memory when the arrival of tags becoming higher per time unit.

In the future, we aim to set the size of landmark window dynamically based on the object’s departure rate from the reader’s vicinity. The benefit of this study is to reduce the probability of false positives. Most of the approaches only related dynamic setting with the object’s arrival rate. Unlike during the arrival, an object does not inform the reader directly that it has left the reading area. Object departure rate is more suitable than the arrival rate in determining the window size dynamically because it indicates which reading can be removed from the window without affecting the quality of data filtering. To test the practical relevance of the proposed duplicate reading detection and removal algorithm, we plan to implement and test the algorithm in actual deployment environments.

## Figures and Tables

**Table 1.** Summary of existing duplicate RFID data filtering approaches.

**Existing Approaches**	**Weaknesses**
ESP and RFID Cube	Delayed process because need to wait all readings to be complete before duplicate readings can be filtered.
RRE	Only suitable if the tagged objects are not moving which is rare in RFID application.
Sliding windows	Need to scan along the sliding windows every time new reading coming in which is not efficient because it scans almost the same data.
Landmark windows	The size of the windows can be very big if want to produce more accurate results than sliding windows.
Bloom filter	It does not allow deletion which can make its filter easily becoming ‘full’ and generate a lot of false positive.
TDMA, MRFID	Put readers into different slot to do readings. It is not suitable for application that requires all the reader to be turned on at the same time.
EIFS	Only do duplicate filtering and does not preserved reading to the rightful reader.

**Table 2.** The condition of CBF after tag 1 is hashed 3 times.

**CBF**	**30**	**30**	**0**	**30**	**0**	**0**
Counter positions	[0]	[1]	[2]	[3]	[4]	[5]

**Table 3.** Reading on tag A1 by different readers.

**Time**	**Reader ID**	**Tag ID**	**Count of readings**
100	R1	1	12
100	R2	1	3
200	R1	2	3
200	R2	2	10
